# Diclofenac versus tramadol for mucositis related pain in head and neck cancer patients undergoing concurrent chemoradiation—a phase 3 study

**DOI:** 10.3332/ecancer.2021.1318

**Published:** 2021-11-18

**Authors:** Amit Joshi, Vijay Maruti Patil, Vanita Noronha, Atanu Bhattacharjee, Nandini Menon, Amit Kumar, Parmanand Jain, Sadaf Mukadam, Avadhoot Shrinivas, Anjali Punia, Anuja Abhyankar, Amit Agarwal, Satvik Khaddar, Anu Rajpurohit, Kanteti Aditya Pavan Kumar, Rahul Ravind, Kishore Das, Vikas Talreja, Sachin Dhumal, Kumar Prabhash

**Affiliations:** 1 Department of Medical Oncology, Tata Memorial Centre, HBNI, Mumbai, 400012, India; 2 Section of Biostatistics, Centre for Cancer Epidemiology, Tata Memorial Centre, Navi-Mumbai, 410210, India; 3 Pain Clinic, Department of Anesthesia, Tata Memorial Centre, HBNI, Mumbai, 400012, India; † These authors contributed equally

**Keywords:** pain, oral mucositis, head and neck, chemoradiation, diclofenac, tramadol

## Abstract

**Background:**

Oral mucositis related pain during CTRT in head and neck cancers is a common problem. Unfortunately, in spite of it being common, there is limited evidence for selection of systemic analgesic in this situation. Hence, this study was designed to compare the analgesic effect of a non-steroidal anti-inflammatory drug (diclofenac) versus a weak opioid (tramadol).

**Patients and methods:**

This was an open-label, parallel design, superiority randomised controlled study. In this study, head and neck cancer patients undergoing radical or adjuvant chemoradiation, who had grade 1 or above mucositis (in accordance with Common Terminology Criteria for Adverse Events version 4.03) and had pain related to it were randomly assigned to either diclofenac or tramadol for mucositis related pain control. The primary endpoint was analgesia after the first dose. The secondary endpoints were the rate of change in analgesic within 1 week, adverse events and quality of life.

**Results:**

One hundred and twenty-eight patients were randomised, 66 in diclofenac and 62 in tramadol arm. The median area under the curve for graph of pain across time after first dose of pain medication for the diclofenac arm and the tramadol arm was 348.936 units (range: 113.64–1,969.23) and 420.87 (101.97–1,465.96), respectively, (*p* = 0.05619). Five patients (8.1%) in the tramadol arm and 11 patients (16.7%) in the diclofenac arm required a change in analgesic within 1 week of starting the analgesic (*p* = 0.184). There was no statistically significant difference in any adverse events between the two arms. However, the rate of any grade of renal dysfunction was numerically higher in the diclofenac arm (10.6% versus 4.8%, *p* = 0.326).

**Conclusion:**

In this phase 3 study, evaluating diclofenac and tramadol for chemoradiation induced mucositis pain, there was no statistical difference in analgesic activity of these two drugs.

## Introduction

Mucositis is common in head and neck cancer patients undergoing concurrent chemoradiation [1]. The overall incidence of any grade mucositis ranges between 65.3% and 96.9%, while the incidence of grade 3 and above mucositis is between 17.3% and 55.8% [1, 2]. Mucositis commonly becomes symptomatic between the second and fourth week of treatment and is associated with considerable pain [3]. Mucositis induced pain leads to decreased oral intake, which in turn impairs the nutritional intake, leads to radiation interruption or dose modification and it negatively impacts the patient’s quality of life [3–5].

Management of mucositis related pain is an important component of the treatment of mucositis [6–8]. Both local and systemic analgesia are required for treatment. However, the literature on analgesia for mucositis related pain is vastly concentrated on the use of local analgesics [9]. Use of local rinse consisting of anaesthetic with diphenhydramine & antacid [10], doxepin mouth rinse [10, 11], amitriptyline mouth rinse [12] and diclofenac mouth rinse [13] are all associated with pain relief. Although commonly used, whether these local rinses are associated with a clinically meaningful decrease in pain is an open question [9, 10, 14]. In practice, systemic adjuvants are commonly used for mucositis related pain [15–17].

The World Health Organization (WHO) pain ladder is commonly used to guide the selection of systemic analgesics [18–20]. Non-steroidal anti-inflammatory drugs (NSAIDs) are the recommended first analgesics in accordance with the WHO ladder. In addition to the analgesic effect, these agents also have an anti-inflammatory property which is an added attraction for their use in mucositis. Weak and strong opioids are other options for the management of mucositis related pain [15]. The choice between diclofenac and tramadol seems obvious; the NSAID would be used initially and then tramadol if analgesia is uncontrolled as per the WHO ladder [18]. However, non-selective NSAIDs like diclofenac have a tendency for causing renal side effects [22, 23]. Cisplatin is the commonest agent used for radiosensitisation in head and neck cancer and can cause derangement in renal function [24]. Further, systemic use of selective-NSAID like celecoxib has failed to relieve mucositis related pain in a phase 3 study [25]. However, diclofenac is a non-selective NSAID which has shown better analgesic properties than celecoxib in a different clinical scenario [26]. Hence, we planned a phase 3 study with the hypothesis that the analgesic effect of systemically administered diclofenac would be better than tramadol in mucositis associated pain.

## Methods

### Study conduct and trial design

This was an open-label, parallel-arm, superiority, pragmatic, phase 3, randomised study conducted at Tata Memorial Centre, Mumbai, India. The study protocol was approved by the Institutional Ethics Committee. The trial was registered prospectively with the Clinical Trial Registry of India (CTRI/2016/09/007302 (Registered on 23/09/2016)). It was conducted in accordance with the guidelines for Good Clinical Practice – International Conference on Harmonisation E6(R2), Declaration of Helsinki and Indian Council of Medical Research guidelines. All patients provided written informed consent prior to participation. The study was funded by an intramural grant from the Tata Memorial Center Research Administration Council (TRAC). The funding agency had no role in design and conduct of the study, collection, management, analysis and interpretation of the data, preparation, review or approval of the manuscript, and decision to submit the manuscript for publication. The study protocol (Version 2.0, Dated 28 February 2016) had no amendments post its initial approval and the investigators adhered to the approved study protocol.

### Participants

Adult (age ≥ 18–70 years) head and neck cancer patients who were undergoing concurrent chemoradiation were invited to participate in the study subject to fulfilment of below-mentioned eligibility criteria. The planned dose of radiation had to be 60 Gy or above and administered in a conventional fashion, 1.8–2 Gy per fraction with radiation delivery 5 days a week. Patients with Eastern Cooperative Oncology Group (ECOG) Performance Status (PS) 0–2, with adequate haematological and liver functions, with mucositis related pain of 1 or more on the visual analogue scale (VAS) were invited for the study. Patients who were already on analgesic or had deranged serum creatinine (>1.5 upper limits of normal) or had an allergy to study medications were excluded.

### Randomisation

Patients were randomised in a 1:1 fashion between the two arms. The random allocation sequence was generated by an independent statistician. The allocation was performed by block stratified randomisation. The factors considered for stratification were the site of the tumour (oral versus others), T grouping (T1-2, T3-4), N grouping (N0-N1, N2-N3) and pain score on VAS (below 5 versus 5 or above). Sixteen strata were generated with each having a block size of 4. The study investigators or coordinators did not have access to the randomisation sheets and it was performed by an independent person.

### Interventions

The study had two interventional arms, arm A and arm B. Patients in arm A received tablet diclofenac for pain relief. Diclofenac was administered in a dose of 50 mg per os (PO) thrice daily (TID). Patients in arm B received tablet tramadol for pain relief. Tramadol was administered in a dose of 50 mg PO TID. All patients in both arms received a local rinse with local anaesthetic (benzocaine 20% w/w) and antacid (aluminium hydroxide, magnesium topical and oxetacaine topical) in addition to the systemic analgesic. The first dose was administered under the supervision of the trial staff. Pain scores at baseline (on VAS) and at 5, 15, 30, 60, 120, 240, 300 and 360 minutes after the first dose of analgesic were noted. The VSA had a minimum score of 0 and a maximum score of 10 represented in a horizontal line with 10 cm separation between 0 and 10. The patients were instructed by the trial staff to plot their scores in accordance with the pain felt where 0 – represented no pain, while 10 – represented maximum bearable pain. The score at each time point was measured using a ruler (line gauge).

Subsequent follow-up were conducted weekly till completion of chemoradiation (CTRT). At each week, blood investigations inclusive of complete blood count, serum creatinine, serum electrolytes (Na, K, Ca, Mg), serum glutamic-oxaloacetic transaminase (SGOT), serum glutamate-pyruvate transaminase (SGPT) and total bilirubin were performed. In addition, pain control, compliance with pain medications and adverse events (in accordance with Common Terminology Criteria for Adverse Events version 4.03) were assessed. Pain and analgesic effect were assessed at each visit as per the VAS. If inadequate pain control was noted, then the patient’s analgesic was changed. The definition of inadequate pain control was the presence of pain of 1 or more on VAS. This change was done with the discussion and concurrence of two clinicians. The pattern of change of analgesic was predefined. In case of inadequate pain control in the diclofenac arm, a weak opioid was added. While in case of inadequate pain control in the tramadol arm, an NSAID was added. In case of subsequent inadequate pain control in any arm after the first change, a strong opioid was considered. Quality of life questionnaire (European Organization for the Research and Treatment of Cancer Quality of Life Questionnaire (EORTC QLQ)-c30 and QLQ-HN-35) was administered to patients at baseline, at each subsequent visit till end of treatment.

### Sample size

Assuming a 5% significance level, with 80% power to detect a clinically meaningful effect size of 0.5 of a standard deviation between the two arms, we required 128 patients (64 patients for each arm) based on the two-sample *t*-test with an equal-variance assumption.

### Outcome assessment

The primary endpoint of the study was analgesia for 6 hours post first dose of studied analgesic.

For quantification of the analgesic effect, an area under the curve (AUC) was plotted with time being represented on the *X-*axis and pain scores on the *Y*-axis. On the *X*-axis, the time points 0, 5, 15, 30, 60, 120, 240, 300 and 360 minutes were represented by values 1, 2, 3, 4, 5, 6, 7, 8 and 9 to avoid overweighting the later time points. The values of *Y*-axis were plotted with the baseline score being considered as 100% and the rest of the scores were adjusted accordingly. The secondary outcomes were to compare the proportion of patients requiring a change in analgesic within 1 week of the start of studied analgesic, adverse events, weight loss and quality of life at the end of treatment.

### Statistical methods

Statistical Package for the Social Sciences version 20 and RStudio version were used for analysis. Intention to treat analysis was performed. Descriptive statistics were performed. Ordinal and nominal variables were expressed in terms of percentages with 95% confidence interval (CI) while continuous variables were expressed in terms of the median with range. Missing data were imputed using the multivariate imputation by chained equations. The AUC for analgesia was calculated using the trapezoidal rule. The AUC was compared between the two arms using the Wilcoxon rank-sum test. The effect size index was calculated for the difference in the AUC between the two arms and interpreted as per Cohen. A *p*-value of 0.05 or below was considered as statistically significant while an effect size of 0.5 or above was considered as clinically significant. Post hoc sensitivity analysis was performed using two methods. First was comparing AUC between the two arms using the Wilcoxon rank-sum test. However, the missing data was tackled using the listwise deletion method (complete data set analysis). The second was comparing the pain scores at different time points between the two arms using Linear Mixed Effect Model. The comparison of change of analgesic within 1 week, adverse events and weight loss was performed using Fisher’s test. The quality of life analysis was performed as per the European Organization for Research and Treatment of Cancer (EORTC) guidelines. The linear mixed effect was performed as an extension of the linear model to compare quality of life domains between the two treatment arms. The data were censored for analysis on 9 July 2019.

## Results

### Baseline characteristics

**Figure 1.**
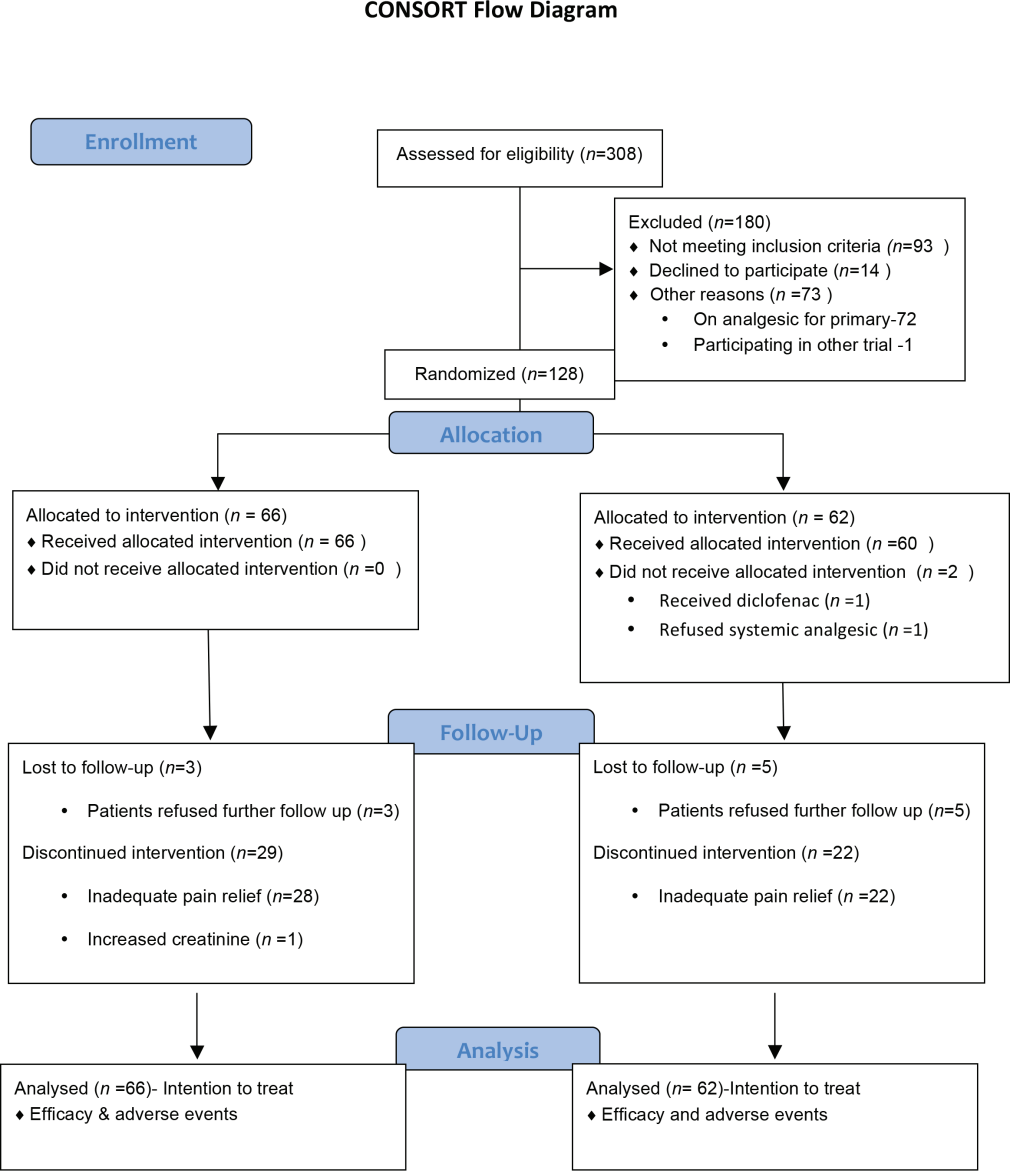
Consort diagram.

The study recruited 128 patients between 26 April 2017 and 20 May 2019, with 66 patients in diclofenac and 62 patients in tramadol arm. Figure 1 shows the consort diagram. The baseline characteristics are shown in Table 1.

### Analgesia within 6 hours

**Figure 2.**
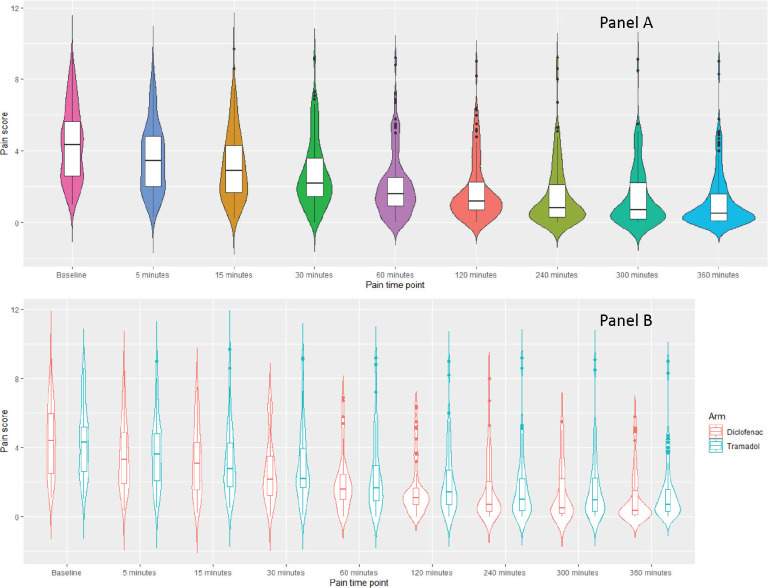
Violin plot depicting the pain scores at baseline at different time points till 6 hours after administration of first dose in both arms.

Pain scores in 8 (6.25%) patients were missing at time points at 5, 15, 30, 120, 240 and 300 minutes. While for 9 (7%) patients, the data of pain scores at 60 and 360 minutes were missing. The pain score at different time points overall and between the two arms is shown in Figure 2 panel A & B, respectively. The median AUC for diclofenac arm and tramadol arm was 348.9 units (range: 113.6–1,969.2) and 420.9 (102.0–1,466.0), respectively, (*p* = 0.06) with the complete dataset method using Wilcoxon rank-sum test. The calculated Cohen effect size for this difference was 0.125, suggesting it was not clinically significant (Supplementary Table 1 and 2). The result of post hoc sensitivity analysis done using listwise deletion method corroborated with the primary analysis. The median AUC for diclofenac and tramadol arm was 365.5 units (range: 113.6–1,969.2) and 420.9 (102.0–1,466.0), respectively, (*p* = 0.11) with the listwise deletion method using Wilcoxon rank-sum test. The sensitivity analysis performed using a linear mixed effect model also confirmed the same findings (Supplementary Table 3). A post hoc analysis of average pain scores over the first 6 hours was also performed using both listwise deletion (*p* = 0.46) and complete dataset methods (*p* = 0.58) and the results were in line with the primary analysis.

### Change of analgesic

**Supplementary Figure 1.**
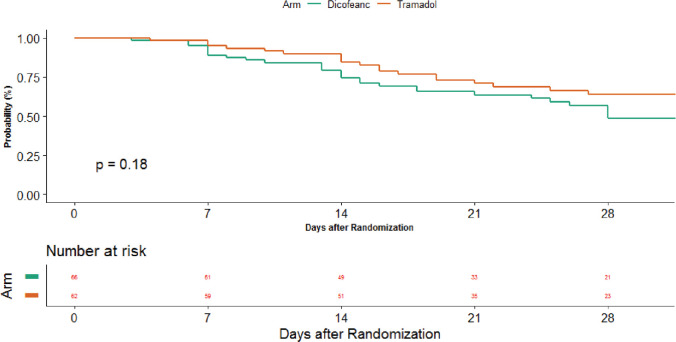
Time to change of analgesic.

Five patients (8.1%) in the tramadol arm and 11 patients (16.7%) in the diclofenac arm required a change in analgesic within 1 week of starting the analgesic (*p* = 0.18) (Supplementary Figure 1). The cause of change in analgesic in all patients was inadequate pain relief. The unadjusted odds ratio for change in analgesic was 2.280 (95% CI: 0.744–6.989) in favour of tramadol arm (*p =* 0.15). The adjusted odds ratio was 2.329 (95% CI: 0.69–7.855) in favour of tramadol arm (*p* = 0.173) (Supplementary Table 2).

Twenty-nine patients (43.9%) in the diclofenac arm and 22 patients (35.5%) in the tramadol arm had a change in studied analgesic till the end of chemoradiation (*p* = 0.37). The cause of change in analgesic in all patients except one was inadequate pain relief. One patient in the diclofenac arm had a rise in serum creatinine and hence the analgesic was changed. The unadjusted odds ratio for change in analgesic was 1.425 (95% CI: 0.699–2.904; *p =* 0.33), while the adjusted odds ratio was 1.612 (95% CI: 0.749–3.471; *p* = 0.222) (Supplementary Table 2). The only factor statistically associated with a higher rate of change of analgesia at the end of treatment was the use of tobacco (odds ratio-3.402; 95% CI: 1.067–10.847, *p* = 0.038). Data are shown in Supplementary Table 2.

The median duration of analgesic in the study was 28.5 days (range: 7–63). The median time to change to analgesic was 28 days (95% CI: 18.248–37.752) for diclofenac versus 54 days (95% CI: 30.617–77.383) for tramadol (*p* = 0.18). Tramadol and morphine were required in the diclofenac arm in 23 (34.8%) and 8 (12.1%) patients, respectively. While diclofenac and morphine were required in 16 patients (25.8%) and 7 patients (11.3%), respectively, in the tramadol arm.

### Adverse events & compliance

The compliance in diclofenac and tramadol arm was observed in 55 (83.3%) and 52 (83.9%), respectively, (*p* = 1). The incidence of nasogastric tube insertion was 54.5% (36) and 56.5% (35) in diclofenac and tramadol arm, respectively, (*p* = 0.86). The adverse events in both arms are shown in Table 2. There was no statistically significant difference in any adverse events between the two arms. However, the rate of any grade renal dysfunction was numerically higher in the diclofenac arm (10.6% versus 4.8%, *p* = 0.33).

### Compliance with cancer-directed treatment

The details of radiation dose planned, technique, chemotherapy planned are shown in Table 3. The radiation technique was imbalanced between both arms with a higher number of patients in the diclofenac arm were treated with three-dimensional conformal radiation therapy (3DCRT) than in the tramadol arm (18.2% versus 1.6%, Table 3). There was also a difference in cumulative dose of cisplatin received between both arms. In the diclofenac arm, 49 patients (80.3%, *n* = 61), while in the tramadol arm 55 patients (93.2%, *n* = 59) received a cumulative dose of cisplatin ≥ 200 mg/m^2^ (*p* = 0.06).

### Quality of life analysis

The quality of life at baseline and at each visit was similar between both arms. The results of quality of life analysis using linear mixed effect models are shown in Supplementary Table 3.

## Discussion

This study evaluated the role of systemic analgesia in mucositis pain. In the current study, there was a decrease in pain scores after administration of the first dose of systemic analgesic (Figure 2); however, there was no statistical difference or clinically relevant difference (as effect size was only 0.125) [27] in the efficacy of the two types of analgesics. Thus, suggesting that both tramadol and diclofenac in the doses used in the current study have a similar analgesic effect within 6 hours of the first dose. It is important for an analgesic not only to have a rapid decrement in pain but also to sustain the analgesia over the required duration. The sustenance of analgesia as suggested by proportion of patients in whom a change in analgesic was required by 1 week and by the end of treatment was also statistically similar between the two arms. Thus, implying that both diclofenac and tramadol have similar analgesic activity.

The selection of any drug, as the drug of choice, rests on adverse event profile if efficacy is similar. The adverse events were similar between both arms and were largely dictated by the adverse events related to cancer-directed therapy. However, there was a numerical difference in the rate of renal dysfunction between the two arms, with the incidence being twice in diclofenac arm than tramadol arm. The rate of renal dysfunction in the current study is overall lower than that reported in western literature and is primarily because of predominant use of weekly cisplatin [28, 29]. Considering that there is no added advantage of diclofenac with respect to analgesia over tramadol, it might be better to avoid diclofenac especially when cisplatin regimen is used for radiosensitisation. Both diclofenac and cisplatin are known to cause renal dysfunction. Cisplatin causes disruption of the S3 segment of the proximal renal tubule [30], while NSAIDs like diclofenac lead to interstitial nephritis [31]. There seems to be an additive effect of the addition of diclofenac to cisplatin on renal dysfunction. In addition, the presence of volume depletion acts as a predisposition for renal dysfunction with both agents [31, 32]. Due to poor intake and increased insensible loss, mucositis pain is common in patients with severe mucositis. There is also evidence to suggest the development of acute kidney injury during chemoradiation in head and neck cancer patients leads to a decrease in survival [24]. Hence, it seems reasonable that NSAID should be avoided for analgesia in such patients.

The focus of research on analgesic treatment of mucositis pain is largely restricted to local analgesics [10, 11, 14]. However, in practice, a large proportion of patients irrespective of use of local analgesics require systemic analgesia including morphine [15, 17]. As was observed in the current study, >10% of patients require morphine by the end of treatment for pain relief. Use of strong opioids like morphine leads to opioid related side effects like nausea, constipation, sedation, dry mouth which can hamper the quality of life [21]. Hence, it is important to have longitudinal data on change of analgesic, especially assessing the requirement of morphine. The requirement of morphine in current study was also similar between both arms.

Mucositis pain is associated with weight loss, delays and noncompliance with anticancer treatment [33, 34]. In the current study, the rate of weight loss and radiation compliance were similar between both arms. However, the proportion of patients receiving a cumulative dose of cisplatin of 200 mg/m^2^ or more was lower in the diclofenac arm. The probable reason for this is due to renal dysfunction caused by diclofenac. A cumulative dose of cisplatin of 200 mg/m^2^ or more is an important factor influencing efficacy outcomes in head and neck cancer [35].

The current study has its strengths and limitations. The strengths of this study are that it is a unique study evaluating the role of commonly prescribed systemic analgesics, provides data regarding compliance, longitudinal change of analgesic and quality of life, which is sparse in literature. The limitations are that the study was a single centre, open label study and there was no placebo arm in this study. Since there was no placebo arm, the impact of diclofenac and tramadol over placebo could not be studied. However, this aspect was considered at the time of conceptualisation of the study and it was considered unethical to deny patients of an analgesic. The study was conducted in a single centre. However, it led to a uniform treatment decision across both arms. The study was an open label study and hence both patients and physicians were aware about the study arm. However, the study methodology required patients to mark the pain on VAS and a change in analgesic was not permitted unless the prespecified VAS score criteria in the protocol were met, thus decreasing the probability of physician bias.

## Conclusion

In this phase 3 study, evaluating diclofenac and tramadol for mucositis pain, analgesic efficacy of both analgesics was found to be similar but diclofenac was associated with a numerically statistically non-significant higher rate of renal dysfunction.

## List of abbreviations

CTRT, Chemoradiation; NSAIDs, Non-steroidal anti-inflammatory drugs; AUC, Area under the curve; CTRI, Clinical Trial Registry of India; WHO, World Health Organization; TRAC, Tata Memorial Center Research Administration Council; ECOG PS, Eastern Cooperative Oncology Group Performance Status; VAS, Visual Analogue Scale; EORTC QLQ, European Organization for the Research and Treatment of Cancer Quality of Life Questionnaire; 3DCRT, Three-dimensional conformal radiation therapy.

## Conflicts of interest

The authors declare the following financial interests/personal relationships which may be considered as potential competing interests:

Dr. Noronha reports grants from Dr. Reddy’s Laboratories Inc., grants from Amgen, grants from Sanofi Aventis, outside the submitted work.

Dr. Prabhash reports grants from Biocon Ltd, grants from Dr. Reddy’s Laboratories Inc., grants from Fresenius Kabi India Pvt Ltd, grants from Alkem Laboratories, grants from Natco Pharma Ltd, grants from BDR Pharmaceuticals Intl Pvt Ltd, grants from Roche Holding AG, outside the submitted work.

None of the other authors have anything to declare that may be considered as potential competing interests.

## Trial registration

Clinical Trials Registry-India (CTRI): CTRI/2016/09/007302 (Registered on 23/09/2016) Trial Registered Prospectively.

## Funding

This work is supported by TRAC (Grant Number - not applicable). The funding agency had no role in design and conduct of the study, collection, management, analysis, and interpretation of the data, preparation, review or approval of the manuscript, and decision to submit the manuscript for publication.

## Figures and Tables

**Supplementary Table 1.** 

	Value	Standard error	Degrees of freedom	P-value	AIC
**Model1**					
Intercept	3.65	0.43	1023	0.00	3818.426
Arm	0.23	0.27	126	0.38	
Visit	-.39	0.01	1023	0.00	
**Model2**					
Intercept	2.09	0.43	1024	0.00	4521.805
Arm	0.23	0.27	126	0.38	

Supplementary Table 1- Result obtained through Linear Mixed Effect Model by maximum likelihood.AIC-Akaike Information Criterion

**Supplementary Table 2.** 

Variable	Type	Odds ratio	95% CI of the odds ratio	P-value
The adjusted odds ratio for change in analgesic at 1 week				
Arm	Binary	2.329	0.69-7.855	0.173
Site	Binary	0.814	0.229-2.896	0.75
T group	Binary	0.728	0.19-2.784	0.643
N group	Binary	1.2227	0.382-3.943	0.731
Baseline pain score	Binary	0.417	0.136-1.281	0.127
Age	Continuous	1.037	0.975-1.103	0.251
Tobacco use	Binary	3.117	0.742-13.603	0.119
The adjusted odds ratio for change in analgesic at end of chemoradiation				
Arm	Binary	1.612	0.749-3.471	0.222
Site	Binary	1.064	0.456-2.478	0.887
T group	Binary	0.737	0.293-1.856	0.517
N group	Binary	1.383	0.636-3.01	0.413
Baseline pain score	Binary	0.863	0.401-1.858	0.707
Age	Continuous	0.99	0.95-1.031	0.621
Tobacco use	Binary	3.402	1.067-10.847	0.038

Supplementary Table 2- Adjusted odds ratio for change in analgesic at 1 week and end of chemoradiation.CI- Confidence interval. Site- Oral(reference) versus non-oral. T group- T1-T2 (reference) versusT3-T4. N group-N0-N1 (reference) versus N2-N3. Baseline pain score- below 5 versus 5-10 (reference). Arm- Tramadol( reference) versus Diclofenac. Age was a continuous variable. Tobacco use- present (reference) versus absent.

**Supplementary Table 3.** 

Domain		Value	Standard error	Degree of freedom	t-value	P-value
Pain (HNPA)	Intercept	41.68	2.48	399	16.75	0.00
	Tramadol	-3.41	3.58	113	-0.95	0.34
Swallowing (HNSW)	Intercept	41.94	2.87	352	14.58	0.00
	Tramadol	-2.26	4.07	110	-0.55	0.57
Speech problems (HNSP)	Intercept	42.53	2.85	354	14.90	0.00
	Tramadol	-2.09	4.07	110	-0.51	0.60
Trouble with social eating (HNSO)	Intercept	36.82	2.96	356	12.42	0.00
	Tramadol	2.22	4.27	112	0.52	0.60
Trouble with social contact (HNSC)	Intercept	35.32	3.55	263	9.94	0.00
	Tramadol	0.45	5.07	98	0.08	0.92
Less sexuality (HNSX)	Intercept	36.82	2.96	356	12.42	0.00
	Tramadol	2.22	4.27	112	0.52	0.60
Teeth (HNTE)	Intercept	35.32	3.55	263	9.94	0.00
	Tramadol	0.45	5.07	98	0.08	0.92
Opening mouth (HNOM)	Intercept	57.64	4.73	151	12.16	0.00
	Tramadol	-7.19	6.48	65	-1.10	0.27
Dry mouth (HNDR)	Intercept	44.96	3.54	107	12.66	0.00
	Tramadol	3.63	4.84	70	0.74	0.45
Sticky saliva (HNSS)	Intercept	53.47	2.76	265	0.00	0.00
	Tramadol	-1.06	4.11	94	-0.25	0.79
Coughing (HNCO)	Intercept	54.55	2.90	275	18.75	0.00
	Tramadol	-3.08	4.16	95	-0.74	0.46
Felt ill (HNFI)	Intercept	56.85	2.80	330	20.25	0.00
	Tramadol	-3.76	3.99	105	-0.94	0.34
Pain killers (HNPK)	Intercept	53.12	2.94	289	18.04	0.00
	Tramadol	-3.05	102	102	-0.72	0.46
Nutritional supplement s (HNNU)	Intercept	52.17	2.86	303	18.21	0.00
	Tramadol	-2.09	4.08	100	-0.51	0.60
Feeding tube (HNFE)	Intercept	33.33	0.34	281	95.64	0.00
	Tramadol	0.95	0.51	103	1.84	0.06
Weight loss (HNWL)	Intercept	33.33	0.63	126	52.13	0.00
	Tramadol	1.56	0.96	67	1.61	0.11
Weight gain (HNWG)	Intercept	33.33	0.67	119	49.06	0.00
	Tramadol	1.99	1.09	54	1.81	0.07
Global health status (QL2)	Intercept	53.65	2.61	377	20.49	0.00
	Tramadol	1.45	3.74	108	0.38	0.69
Physical functioning (PF)	Intercept	73.35	2.13	441	34.37	0.00
	Tramadol	-1.40	3.04	115	-0.45	0.64
Role functioning (RF)	Intercept	80.09	2.48	420	32.25	0.00
	Tramadol	-0.73	3.53	114	-0.20	0.83
Emotional functioning (EF)	Intercept	70.01	2.54	429	27.51	0.00
	Tramadol	0.27	3.63	114	0.07	0.93
Cognitive functioning (CF)	Intercept	80.11	2.40	433	33.31	0.00
	Tramadol	-0.30	3.43	115	-0.08	0.92
Social functioning (SF)	Intercept	80.30	2.43	405	32.96	0.00
	Tramadol	-4.70	3.49	114	-1.34	0.18
Fatigue (FA)	Intercept	43.69	2.45	391	17.78	0.00
	Tramadol	-1.78	3.52	114	-0.50	0.61
Nausea and vomiting (NV)	Intercept	31.87	2.40	268	13.23	0.00
	Tramadol	3.86	3.32	98	1.16	0.24
Pain (PA)	Intercept	40.85	2.49	357	16.37	0.00
	Tramadol	-1.85	3.58	112	-0.51	0.60
Dyspnoea (DY)	Intercept	40.49	2.52	140	16.04	0.00
	Tramadol	4.92	3.72	74	1.32	0.18
Insomnia (SL)	Intercept	48.71	2.728	198	17.85	0.00
	Tramadol	-0.75	4.02	93	0.18	0.85
Appetite loss (AP)	Intercept	51.87	2.63	294	19.68	0.00
	Tramadol	0.00	3.70	104	0.00	0.99
Constipatio n (CO)	Intercept	46.08	2.68	178	17.15	0.00
	Tramadol	-1.47	3.83	86	-0.38	0.70
Diarrhoea (DI)	Intercept	41.41	3.48	94	11.88	0.00
	Tramadol	7.55	7.55	67	1.55	0.12
Financial difficulties (FI)	Intercept	57.65	3.29	230	17.48	0.00
	Tramadol	-1.90	4.65	94	-0.40	0.68

Supplementary Table 3- Quality of life analysis. The results are represented with diclofenac arm being considered as standard in relation with tramadol arm.

**Table 1.** Baseline characteristics.

Variable	Diclofenac arm (*n* = 66)	Tramadol arm (*n* = 62)
Age-Median (range) in years	52.5 (28–70)	48 (28–70)
Gender-no (%) Male Female	54 (81.8) 12 (18.2)	52 (83.9) 10 (16.1)
ECOG PS-no (%) 0–1 2	65 (98.5) 1 (1.5)	60 (96.8) 2 (3.2)
Tumour site-no (%) Oral Oropharynx Larynx Hypopharynx Others	29 (43.9) 13 (19.7) 10 (15.2) 13 (19.7) 1 (1.5)	24 (38.7) 21 (33.9) 8 (12.9) 9 (14.5) -
T grouping-no (%) T1-T2 T3-T4	10 (15.1) 56 (84.9)	18 (29.1) 44 (70.9)
N grouping-no (%) N0-N1 N2-N3	33 (50) 33 (50)	29 (46.8) 33 (53.2)
Stage grouping-no (%) Stage III Stage IV	22 (33.3) 44 (66.7)	22 (35.5) 40 (64.5)
Indication for radiation-no (%) Definitive Adjuvant	38 (57.6) 28 (42.4)	41 (66.1) 21 (33.9)
Pain score at baseline-no (%) 1–<5 5–10	31 (62.1) 25 (37.9)	38 (61.3) 24 (38.7)
Comorbidities-no (%) Hypertension Diabetes mellitus	7 (10.6) 8 (12.1)	8 (12.9) 5 (8.1)
Habits-no (%) Tobacco-oral Tobacco-smoke Alcohol	43 (65.2) 23 (34.8) 8 (12.1)	41 (66.1) 19 (30.6) 5 (8.1)

ECOG, Eastern Cooperative Oncology Group; PS, Performance status

**Table 2.** Adverse event details in both arms. The numbers with percentages in brackets are depicted.

Adverse event	Diclofenac arm (*n* = 66)		Tramadol arm (*n* = 62)		*p* value for any grade events	*p* value for grade 3 & above events
	Any grade	Grade 3–5	Any grade	Grade 3–5		
Mucositis	66 (100)	23 (34.8)	62 (100)	17 (27.4)	-	0.47
Dysphagia	60 (90.9)	20 (30.3)	56 (90.3)	12 (19.4)	1	0.22
Weight loss	39 (59.1)	1 (1.5)	39 (62.9)	-	0.72	1
Nausea	26 (39.4)	2 (3)	20 (32.3)	2 (3.2)	0.47	1
Vomiting	17 (25.8)	-	18 (29)	2 (3.2)	0.7	0.23
Constipation	7 (11.3)	-	13 (19.7)	-	0.23	-
Rise in creatinine	7 (10.6)	-	3 (4.8)	-	0.33	-
Hyponatraemia	60 (90.9)	17 (25.8)	54 (87.1)	17 (27.4)	0.58	0.84
Hypokalaemia	4 (6.1)	1 (1.5)	8 (12.9)	3 (4.8)	0.23	0.35
Hypomagnesaemia	22 (33.3)	-	28 (45.2)	1 (1.6)	0.21	0.48
SGOT rise	9 (13.6)	1 (1.5)	11 (17.7)	1 (1.6)	0.63	1
SGPT rise	12 (18.2)	1 (1.5)	14 (22.6)	1 (1.6)	0.66	1
Anaemia	53 (80.3)	3 (4.5)	54 (87.1)	-	0.35	0.25
Neutropenia	15 (22.7)	6 (9.1)	21 (33.9)	6 (9.7)	0.17	1
Thrombocytopenia	14 (21.2)	2 (3)	15 (24.2)	1 (1.6)	0.83	1

SGOT, Serum glutamic-oxaloacetic transaminase; SGPT, Serum glutamic pyruvic transaminase

**Table 3.** Chemoradiation compliance details.

Variable	Diclofenac arm (*n* = 66)	Tramadol arm (*n* = 62)	*p* value
Technique-no (%) Conventional 3DCRT Intensity modulated radiotherapy	40 (60.6) 12 (18.2) 14 (21.2)	48 (77.4) 1 (1.6) 13 (21)	0.005
Planned dose in Gy-no (%) 60 >60–<70 70	28 (42.5) 9 (13.6) 29 (43.9)	22 (35.5) 5 (8.1) 35 (56.4)	0.268
Chemotherapy planned-no (%) 3 weekly cisplatin Weekly cisplatin Cisplatin-nimotuzumab Carboplatin Nimotuzumab Docetaxel	- 52 (78.9) 9 (13.6) 3 (4.5) - 2 (3)	4 (6.5) 48 (77.4) 7 (11.3) 1 (1.6) 1 (1.6) 1 (1.6)	0.119
Planned radiation dose not completed-no (%)	4 (6)	2 (3.2)	1
Reasons for non-completion-no (%) Default Progression Adverse event	2 (3) 1 (1.5) 1 (1.5)	1 (1.6) - 1 (1.6)	-
Completed chemotherapy Yes No-adverse events No-patient refused	55 (83.3) 7 (10.6) 4 (6.1)	49 (79) 10 (16.1) 3 (4.8)	0.69
200 mg/m^2^ of cisplatin received-no (%)	49 (80.3)^a^	55 (93.2)^a^	0.058

a *n* used for the percentage calculation was 61 and 59 in diclofenac and tramadol arm, respectively. This was the number of patients who received cisplatin in both arms
